# Supplementary material for: Melatonin and Its Metabolites Can Serve as Agonists on the Aryl Hydrocarbon Receptor and Peroxisome Proliferator-Activated Receptor Gamma
Source: Int J Mol Sci. 2023 Oct 23;24(20):15496. doi: 10.3390/ijms242015496 (PMC10607054; doi:10.3390/ijms242015496)
Supplement: Supplementary file 1 [file ijms-24-15496-s001.zip › ijms-2600395-supplementary.pdf]

Melatonin and its metabolites can serve as agonists on the AhR and PPARγ

Andrzej T. Slominski[1*#], Tae-Kang Kim[1*], Radomir M. Slominski[1,2*], Yuwei Song[1, 2*], Shariq Qayyum[1,3], Wojciech Placha[4], Zorica Janjetovic[1], Konrad Kleszczynski[5], Venkatram Atigadda[1], Yuhua Song[6], Chander Raman[1], Cornelis J. Elferink[7], Judith Varady Hobrath[8]Anton M. Jetten[9], Russel Reiter[10]

[1]Department of Dermatology, [2]Department of Genetics, [6]Department of Biomedical Engineering, UAB, Birmingham, AL, USA
[3]Brigham's Women Hospital and Harvard University, Boston, MA, USA
[4]Department of Medicinal Biochemistry, Collegium Medicum, Jagiellonian University, Krakow, Poland
[5]Department of Dermatology, University of Munster, Munster, Germany
[7]Department of Pharmacology and Toxicology, University of Texas Medical Branch, Galveston, TX, USA
[8]Sygnature Discovery, Biocity, Nottingham NG1 1GF, United Kingdom
[9]Cell Biology Section, NIEHS, National Institutes of Health, Research Triangle Park, NC, USA
[10]Department of Cell Systems and Anatomy, UT Health, Long School of Medicine, San Antonio, TX 78229, USA.

Table S1. Glide XP docking scores of compounds docked into the AhR homology model

Compounds Identification	Docking Score
**Control**	
Indirubin	-9.56
Indole acetic acid	-9.03
**melatonin and selected metabolites**	
N-acetyl serotonin	-9.37
N-acetyl-5-methoxykynuramine	-9.33
Indole 3-carbinol	-9.22
Serotonin	-8.94
Melatonin	-8.76
2-Hydroxy-melatonin	-8.70
N-Acetyl-N-formyl-5-methoxykynuramine	-8.65
6-Hydroxy-melatonin	-8.59
CysteinylDOPA	-8.58
5-Methoxyindole acetic acid	-8.43
5-Methoxytryptamine	-8.36
4-Hydroxy-melatonin	-8.19
Dihydroxyindole carboxylic acid	-8.17
5-Methoxytryptophol	-8.05
Dihydroxyindole	-8.00
L-Tryptophan	-7.92
L-DOPA	-7.48
GlutathionylDOPA	-6.99
2-CysteinylDOPA	-6.65

Table S2. Glide XP docking scores of compounds docked into the PPARγ homology mode

Compounds Identification	Docking Score
**Control**	
rosiglitazone	-8.20
**melatonin and selected metabolites**	
2-GlutathionylDOPA	-10.19
2-Hydroxy-melatonin	-9.54
Indole acetic acid	-9.03
N-Acetyl-N-formyl-5-methoxykynuramine	-8.98
Indirubine	-8.92

GlutathionylDOPA	-8.85
CysteinylDOPA	-8.80
5-Methoxyindole acetic acid	-8.74
5-Methoxytryptophol	-8.73
4-Hydroxy-melatonin	-8.69
6-Hydroxy-melatonin	-8.54
Dihydroxyindole carboxylic acid	-8.40
Dihydroxyindole	-8.37
2-CysteinylDOPA	-8.20
N-acetyl-5-methoxykynuramine	-7.83
Melatonin	-7.77
Serotonin	-7.74
Indole 3-carbinol	-7.51
5-Methoxytryptamine	-7.38
L-Tryptophan	-7.07
L-DOPA	-6.73

Table S3. Summary of AhR residues forming the binding site region of Melatonin, 6(OH)Melatonin, 5-MTT, AFMK in docked complexes.

Compounds	Polar or charged residues	Non-polar residues
Melatonin	Y322, C333, S336, H337, S365, Q383	F295, G321, I325, M340, M348, F351, L353, A367, V381
6(OH)Melatonin	H291, S320, Y322, C333, H337, Q383	L315, G321, F324, I325, A334, L353
5-MTT	H291, Y322, C333, S336, H337, S346, S365	F295, M340, G347, M348, F351, A367, I379, V381
AFMK	S320, Y322, C333, S336, H337, S365, Q383	F295, G321, F324, G347, M348, F351, L353, A367, V381

Table S4. Summary of PPARγ residues forming the binding site region of Melatonin, 6(OH)Melatonin, 5-MTT, AFMK in docked complexes.

Compounds	Polar or charged residues	Non-polar residues
Melatonin	R288, E295, S342, E343	F226, P227, L228, A292, I296, I325, I326, M329, L330, L333, V339, L340, I341, G346
6(OH)Melatonin	C285, R288, S289, H323, Y327, K367, H449	A292, I326, I326, M329, L330, L333, F363, M364
5-MTT	T229, K230, C285, R288, S289, E295, S332	F226, L228, A233, A292, I326, M329, L330, L333
AFMK	C285, Q286, R288, S289, H323, Y327, K367, H449	L228, F282, I326, M329, L330, L333, V339, L340, I341, F363, M364

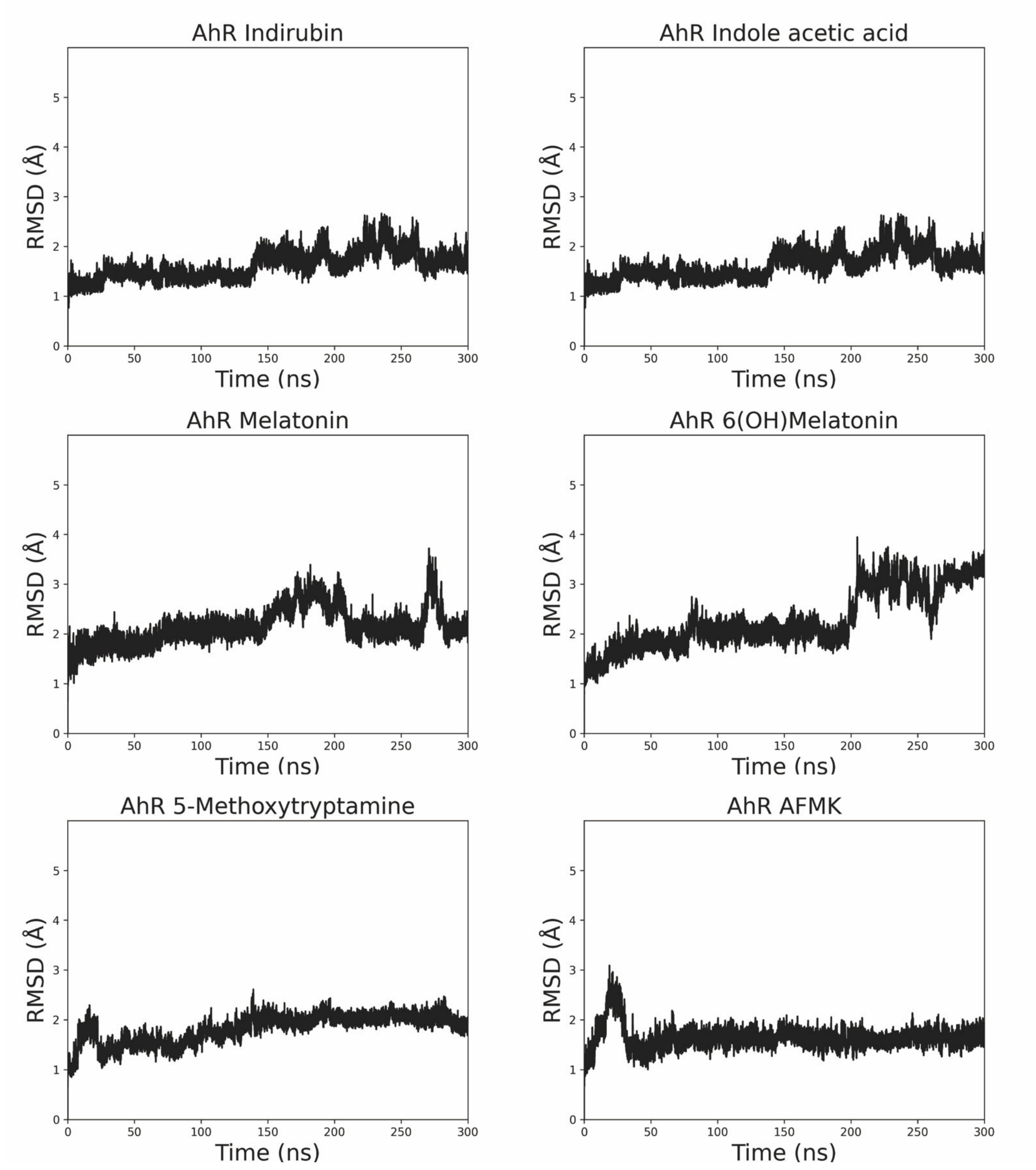

Figure S1. Root mean square deviation (RMSD) showing equilibration of the six MD simulation systems for the natural ligands and melatonin and metabolites with the AhR receptor
